# Outcomes after curatively intended treatment of limited peritoneal metastases and thermal ablation for liver metastases from colorectal cancer

**DOI:** 10.1515/pp-2023-0015

**Published:** 2023-07-24

**Authors:** Rogini Balachandran, Mette Møller Sørensen, Jonas Amstrup Funder, Anders Riegels Knudsen, Lene Hjerrild Iversen

**Affiliations:** Department of Surgery, Aarhus University Hospital, Aarhus, Denmark; Department of Clinical Medicine, Aarhus University, Aarhus, Denmark

**Keywords:** colorectal cancer, cytoreductive surgery, hyperthermic intraperitoneal chemotherapy, liver metastases, peritoneal metastases, radiofrequent ablation

## Abstract

**Objectives:**

Peritoneal metastases (PM) and liver metastases (LM) are present simultaneously in up to 2 % of patients at the time of their colorectal cancer (CRC) diagnosis. Curatively intended treatment includes cytoreductive surgery (CRS) and hyperthermic intraperitoneal chemotherapy (HIPEC) combined with LM resection. A less invasive treatment for LM is ablation. We aimed to estimate overall survival (OS), disease-free survival (DFS) and postoperative data in patients managed simultaneously with CRS, HIPEC and radiofrequency ablation (RFA) as first choice.

**Methods:**

This was a retrospective national cohort study. All patients were treated at Aarhus University Hospital; the only CRS+HIPEC centre in Denmark. We included CRC patients managed with curative intent for simultaneously diagnosed PM and LM in the period January 2016 – December 2021. LM was treated with RFA as first choice, if possible. Survival was calculated by the Kaplan-Meier method.

**Results:**

A total of 25 patients were included, the median age was 60 years (range 43–75 years) and 15 (60 %) were females. The median peritoneal cancer index was 7 (range 0–12), the median number of LM was 1 (range 1–3). Ablation was performed as the only treatment for LM in 18 (72 %) patients. After a median follow-up time of 17.1 months (range 4–36 months), the median OS was 28.6 months (95 % confidence interval (Cl) 15.8;36.1), 1-year OS was 84.0 % (95 % Cl 62.8;93.7). Median DFS was 6.1 months (95 % Cl 4.0;10.3). Median LOS was ten days (range 5–26 days). Both 30-day and 90-day mortality were 0 %.

**Conclusions:**

The selected treatment modality (RFA) for CRC patients with both LM and PM was safe. However, DFS was low. Further research is warranted to investigate if RFA is as effective as LM resection.

## Introduction

The peritoneum and liver are the most common metastatic sites in colorectal cancer (CRC). At the time of diagnosis, peritoneal metastases (PM) are present in 3–6 % [1], [2], [3] and liver metastases (LM) in up to 17 % of patients [1, 4]. The presence of PM and LM decreases overall survival (OS) depending on the extent of disease and how aggressive the offered treatment is. For patients with CRC-PM treated with cytoreductive surgery (CRS) and hyperthermic intraperitoneal chemotherapy (HIPEC), the 5-year OS is 22–43 % [5, 6]. For patients treated only with systemic chemotherapy, 5 year OS is less than 5 % [7]. For patients undergoing resection for CRC-LM, 5 year OS has been reported to fall in the 50–60 % range [8], [9], [10], [11]. For patients undergoing ablation (radiofrequency (RFA) or microwave ablation (MWA)) treatment of LM, the literature has reported lower [12, 13] or comparable 5 year OS [14, 15] compared with resection.

In patients with CRC, simultaneous presence of PM and LM is observed in up to 2 % of cases, at the time of diagnosis [16]. When treated with systemic chemotherapy, the median OS for CRC patients with simultaneous PM and LM has been reported to be five months. In comparison, for patients simultaneously undergoing CRS and HIPEC for PM and surgical resection for LM, median survival increases to 24.4 months [17]. OS is strongly associated with the extent of PM (measured by the Peritoneal Cancer Index (PCI)) and with the number of LM. OS decreases when PCI >7 and with >3 LM [17]. Furthermore, larger LM size has been identified as a poor prognostic factor [18]. The combined surgical treatment of CRC-PM and CRC-LM is a subject of controversy [2, 19, 20]. However, a meta-analysis from 2021 demonstrated that adding parenchymal liver resection to CRS and HIPEC in case of metastatic CRC did not significantly increase major morbidity and mortality [21].

Traditionally, CRC patients with combined PM and LM have undergone surgery for the management of LM. A less invasive treatment modality for LM is ablation. The Danish CRC guidelines [22] for patients with both PM and LM recommend RFA in case of ≤3 curable LM and provided the size of LM is <3 cm. Two recent reviews reporting on OS and disease-free survival (DFS) following combined treatment for patients with CRC-PM and CRC-LM primarily reported on treatment with surgical LM resection. A few studies in these reviews reported on RFA but in combination with resection [21, 23]. The literature is very sparse on curatively intended surgery for CRC-PM combined with RFA for CRC-LM.

In this study, we examined the outcome of patients with CRC-PM treated with CRS and HIPEC primarily combined with RFA for LM. We aimed to report OS, DFS and postoperative data.

## Materials and methods

### Design and setting

A national retrospective cohort study was conducted with data on patients prospectively registered in a local database. Aarhus University Hospital (AUH) is the only centre in Denmark performing CRS and HIPEC. Denmark has roughly 5.8 million inhabitants. Approximately 80 patients undergo CRS and HIPEC annually. Danish healthcare is free and tax financed.

### Patient cohort

We included patients operated with curative intent in the period from January 2016 to December 2021. PM and LM were diagnosed simultaneously. However, surgery for PM and treatment for LM did not need to be performed simultaneously.

According to Danish guidelines [22], CRS and HIPEC for CRC-PM is offered to patients with a physiological age ≤75 years, ≤3 curable LM (each ≤3 cm in size), ≤2 curable lung metastases and no other distant metastases (except for abdominal wall metastases). In simultaneous PM and LM, PCI should be <12. Patients with CRC-PM are typically offered upfront surgery without preoperative chemotherapy. However, there may be exceptions for patients who have recently undergone open resection for their CRC. These patients receive approximately three months of systemic chemotherapy prior to CRS and HIPEC.

Before surgery, all patients undergo a (PET)-CT of the thorax, abdomen and pelvis, a colonoscopy (unless performed ≤6 months ago) and evaluation at a multidisciplinary team (MDT) conference counting CRS surgeon(s), a pathologist, an oncologist and radiologists.

The individual strategy for LM is decided at a liver MDT conference. LM was managed by RFA as first-choice treatment unless contraindications for RFA were observed. Contraindications for RFA treatment of LM in Danish national guidelines [24] are 1) close proximity to centrally located bile ducts, 2) intrahepatic bile duct dilatation, 3) anteriorly located exophytic tumour, 4) need for bilioenteric anastomosis and 5) non-treated/unmanageable coagulopathy. In these cases, a resection or a combination of ablation and resection was conducted.

### Operative procedures and postoperative course

CRS was performed according to a standardised protocol with removal of all macroscopic disease as described by Sugarbaker [25]. The extent of PM was quantified by PCI [26], [27], [28].

In extension of CRS, HIPEC was performed using open technique. The chemotherapy used was oxaliplatin (260 mg/m^2^) combined with intravenous 5-fluorouracil and leucovorin [29], and perfusion time was 30 min. In case of severe neurotoxicity from previous oxaliplatin treatment or in case patients had developed PM shortly after receiving adjuvant oxaliplatin-based chemotherapy, mitomycin C (35 mg/m^2^) was used with a perfusion time of 90 min.

Each patient was postoperatively admitted for approx. 7–10 days at the AUH and then discharged or transferred to a local hospital. According to our guideline, until 2017 patients were to be hospitalised for a minimum of ten days. From 2017, mandatory length of hospital stay (LOS) was reduced to seven days. After surgery, all patients were referred to the Medical Oncology Department for final decision on whether to receive adjuvant chemotherapy.

### Follow-up

The patients underwent follow-up with (PET)-CT of the thorax, abdomen and pelvis at 3, 6, 12, 18, 24 and 36 months after surgery. Additionally, CT of the liver was performed one month after RFA.

### Outcomes

The primary outcome was OS. Secondary outcomes included DFS, recurrence (date and site), LOS, complication rate (Clavien-Dindo grade ≥3) [30, 31], 30 day and 90 day mortality.

Recurrence was defined as a relapse of the disease by recurrence of PM, LM or any other extraperitoneal metastases. Recurrence should be detected by imaging ((PET)-CT) and/or histopathology.

### Data collection

The following data were collected: age, gender, American Society of Anesthesiology (ASA) score, cancer site, preoperative (radio-)chemotherapy, PCI, chemotherapy administered during HIPEC, timing of treatment for LM compared with CRS and HIPEC, LM treatment provided, number of LM, LOS and postoperative complications within 30 days. Mortality was registered up to 90 days. If patients were transferred from ours to another hospital before final discharge, we registered these variables for as long as the patient was admitted to our hospital.

Data on date of recurrence, recurrence site and vital status were collected from the medical records, which are updated daily from the Danish Civil Registration System [32].

The study was approved by the Danish Patient Safety Authority (ref: 31-1521-311).

### Statistical analysis

Descriptive analysis was undertaken to report variable frequencies. Follow-up time was calculated from the date of CRS and HIPEC surgery until death or end of follow-up (31 October 2022). OS and DFS were estimated by the Kaplan-Meier method. OS was calculated from the date of CRS and HIPEC to either the date of death or to 31 October 2022, whichever occurred first. DFS was calculated from the date of CRS and HIPEC to the date of diagnosed recurrence or to 31 October 2022, whichever occurred first. Patients were considered censored if still alive on 31 October 2022 or if they died of other causes than cancer.

STATA 15 (STATA^®^, release 15 IC; StataCorp LLC, College Station, TX, USA) was used for data analysis.

## Results

In total, 25 CRC patients underwent curatively intended surgery for PM combined with treatment for a simultaneously diagnosed LM in the period from January 2016 to December 2021. Patient demographics are presented in Table 1. Females constituted 15 (60 %), and median age was 60 years (range 43–75 years). Most patients underwent CRS and HIPEC due to colon cancer (80 %), and 52 % underwent CRS and HIPEC for synchronously diagnosed PM. The median number of PCI was seven (range 0–12). Almost two thirds of the patients (64 %) underwent upfront surgery, i.e., without preoperative chemotherapy. The chemotherapy administered during HIPEC perfusion was oxaliplatin for 15 patients (60 %), whereas mitomycin C was used for the remaining patients. The median number of LM was one (range 1–3). Most patients, 16 (64 %), were treated with RFA only for their LM. Five patients (20 %) underwent resection, two patients underwent RFA and resection and two patients were ablated with MWA. The majority of patients, 21 (84 %), underwent treatment for LM simultaneously with CRS and HIPEC. In the remaining four, one patient underwent RFA eight days prior to CRS and HIPEC, and one patient underwent RFA and resection 45 days prior to CRS and HIPEC. The last two patients underwent resection for their LM, followed by systemic chemotherapy and then CRS and HIPEC were performed. The majority of patients (84 %) were referred for adjuvant systemic chemotherapy at their local hospital and 24 % completed the chemotherapy. For 14 (56 %) patients, we lacked data on whether they received systemic chemotherapy or not.

**Table 1:** Demographics of CRC patients undergoing combined treatment for PM and LM.

	Number of patients n=25
Gender	
Male	10 (40)
Female	15 (60)
Age (median)	60 (43–75)
ASA score	
1	7 (28)
2	15 (60)
3	3 (12)
Tumour origin	
Colon cancer	20 (80)
Rectal cancer	5 (20)
Timing of metastases	
Synchronous	13 (52)
Metachronous	12 (48)
Preoperative chemotherapy	
No	16 (64)
Yes	19 (36)
Preoperative radiotherapy	
No	24 (96)
Short course	1 (4)
Long course	0 (0)
PCI score (median)	7.0 (0–12)
Chemotherapy administered in HIPEC	
Oxaliplatin	15 (60)
Mitomycin C	10 (40)
Timing of treatment for liver metastases	
Simultaneous during CRS and HIPEC	21 (84)
Prior to CRS and HIPEC	4 (16)
Treatment of liver metastases	
RFA	16 (64)
Liver resection	5 (20)
RFA and liver resection	2 (8)
MWA	2 (8)
Number of liver metastases (median)	1 (1–3)
Postoperative adjuvant chemotherapy	
Referred	21 (84)
Received and completed	6 (24)
Received, but completed prematurely	1 (4)
Missing data	14 (56)

Proportions are presented as percentages. Median values are presented as ranges. RFA, radiofrequency ablation; MWA, microwave ablation.

### Overall survival

**Figure 1:**
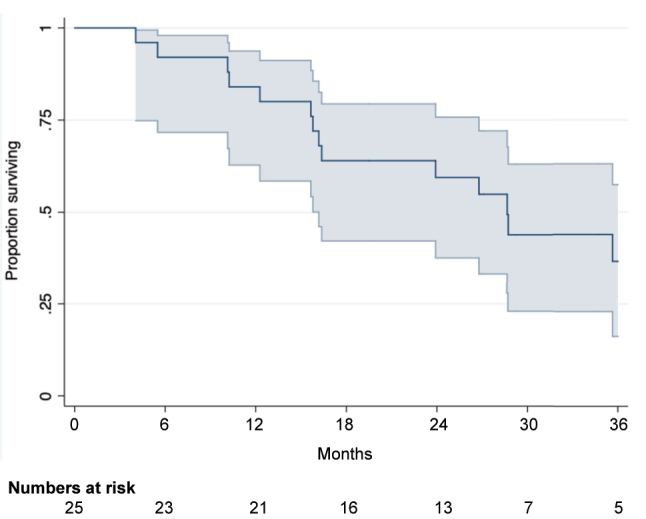
Kaplan Meier survival curve for CRC patients undergoing combined treatment for PM and LM.

The median follow-up time was 17.1 months (range 4–36 months). Median OS was 26.8 months (95 % Cl 15.8;35.1), Figure 1. Based on the Kaplan-Meier analysis, 1 year survival was 84 % (95 % Cl 62.8;93.7), 2-year survival 59.4 % (95 % Cl 37.6;75.8) and 3-year 36.6 % (95 % Cl 16.2;57.4). All deaths were disease related.

### Disease-free survival and recurrence

**Figure 2:**
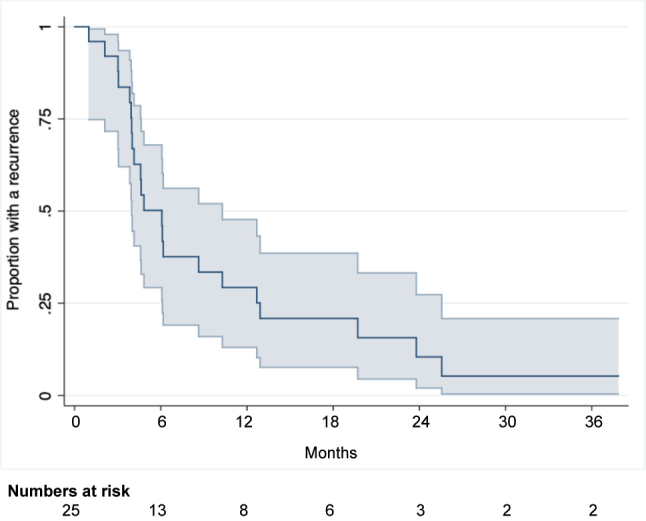
Disease-free survival for CRC patients undergoing combined treatment for PM and LM.

The majority, 22 patients (88 %), developed a recurrence within the follow-up period. Recurrence was diagnosed by imaging ((PET)-CT). For two patients, the recurrence was also proven by histology. For one patient, the exact date of the recurrence diagnosis was unknown, and thus set as the latest date the patient was registered as being disease free according to the medical record. The median DFS was 6.1 months (95 % Cl 4.0;10.3), see Figure 2. At 12 months, 29.3 % (95 % Cl 13.1;47.7) of the patients were without a recurrence. At 18 and 24 months, this proportion was 20.9 % (95 % Cl 7.6;38.6) and 10.5 % (95 % Cl 2.0;27.3), respectively.

Twelve patients (48 %) developed oligometastatic recurrence with the liver being the most frequent recurrence site, occurring in eight patients (36.4 %). Two patients developed PM, one patient had lung metastases and one patient had splenic metastases. Furthermore, four patients developed metastases both in the liver and the lungs, one patient had PM and LM and one patient had PM and metastases in the abdominal wall. Three patients had recurrence in multiple locations. For one patient, we were unable to identify the site of recurrence via the medical records. Recurrence of LM, either as the only recurrence site or as part of multifocal recurrence, was seen in 17 patients (68 %). Among these 17 patients, five (29 %) had a recurrence in a previously RFA-treated cavity. Among the patients who were treated with RFA and resection (two patients) or only resection (five patients), four patients had a recurrence in their liver. These, however, were not located near the previously resected/ablated area.

### Management of recurrent disease

Among the 22 patients who developed recurrence, eight (36 %) repeatedly received curatively intended treatment: Five patients (23 %) underwent another surgical treatment, whereas three patients (14 %) underwent RFA treatment. Palliative systemic chemotherapy was offered to 13 (59 %) patients. For one patient who was referred for treatment of a recurrence at a local hospital, we were unable to gather data.

### Length of hospital stay

The median LOS at our hospital was ten days (range 5–26), see Table 2. Five patients were transferred to their local hospital following a median ten-day LOS at our hospital (range 5–14 days).

**Table 2:** Secondary endpoints – LOS, complication rate and mortality.

LOS at our hospital (median)	
Patients without transfer to another hospital, n=20	11 (6–26)
Patients with transfer to another hospital, n=5	10 (5–14)
Overall, LOS	10 (5–26)
Number of patients with Clavien Dindo 3–4 complications	7 (28 %)
Type and number of Clavien-Dindo 3 complication	9
Blowout of the rectal remnant	1
Pelvic abscess	1
Intraabdominal abscess	1
Fascial dehiscence	2
Pleural effusion requiring chest tube	3
Insertion of gastroenteric tube and feeding tube due to ventricle retention	1
Type and number of Clavien-Dindo 4 complication	3
Hypovolemia and sepsis requiring ICU admission	1
Respiratory failure requiring ICU admission	1
Atrial fibrillation requiring ICU admission	1
Mortality	
30 day mortality	0 %
90 day mortality	0 %

Proportions are presented as percentages. Median values are presented as ranges.

### Postoperative complications

Seven patients (28 %) developed severe postoperative complications (Clavien-Dindo grade ≥3) within 30 days. For five patients, we had no information on postoperative complications developed following transfer to their local hospital. However, all five patients were contacted at the three-month follow-up and no further postoperative complications were reported following transfer. All patients with a postoperative complication recovered. No postoperative mortality occurred within 90 days, Table 2.

## Discussion

In this study, CRC patients undergoing curatively intended CRS and HIPEC for PM combined with RFA as first-choice treatment for LM had a median OS exceeding two years. Furthermore, we report a 1 year survival of 84 % and a 3 year survival of 22 %.

The OS reported in this study is in line with the OS reported in two recent reviews from 2021, reporting on 14 studies [21] and 20 studies [23]. These reviews reported a wide range in median OS, from 15.0–45.1 months [21, 23]. Both reviews differ from our study in some areas. Treatment for LM was mostly surgical resection, whereas in our study RFA was first-choice treatment. One review reported on studies in which CRS and HIPEC and liver resection were performed simultaneously [21]. None of the studies included in the review adopted a two-staged approach, meaning performing CRS, HIPEC and liver resection, as two separate surgeries. In our study, a simultaneous approach was also used in the majority of patients, but 16 % of our patients underwent treatment for LM prior to CRS and HIPEC. This is due to the surgical setting in Denmark with only one centre performing CRS and HIPEC and four centres performing liver surgeries. Lo Dico et al. reported that the sequence of surgery has an impact on survival. Patients with a liver-first approach had a better OS than patients treated with CRS and HIPEC first or patients who had a one-step procedure [33]. Also, the reviews differ in reporting a wide range in mean/median PCI (6.0–18.5) [21, 23]. PCI is a well-recognised prognostic factor for oncological outcome [6, 34]. We reported a median PCI of 7. The HIPEC chemotherapy used and reported in these reviews also varies; mitomycin C, oxaliplatin, irinotecan and cisplatin used as sole agents or in combinations. In our study, patients were treated solely with oxaliplatin (first choice) or mitomycin C. More than one third (36 %) of our patients with a recurrence were offered repeated treatment with curative intent for their recurrence. This may potentially further improve their survival.

We reported a median DFS of six months. Median DFS for patients following combined surgery for PM and LM has been reported to be 8.5 months with up to 86.1 % of the patients having a recurrence within the median follow-up time of 21.8 months [17]. The DFS reported in our study is similar to the lower end of the range of the median DFS reported in one of the previously mentioned reviews [21]. In that review, the DFS ranged from 6.7 to 24.0 months. Importantly, the majority of studies in the review from 2021 reported a median DFS comparable to our finding, and only two studies reported a much higher median DFS of 21 months [35] and 24 months [36]. The study reporting a median DFS of 21 months differs from our study as all patients received systemic chemotherapy before surgery and 66.6 % of the patients received neoadjuvant monoclonal antibodies [35]. Patients whose disease progress during preoperative chemotherapy will not proceed to surgery. Hence, the patients who are offered surgery following preoperative chemotherapy may potentially represent patients with less aggressive disease. In our study, only 36 % received preoperative chemotherapy. In the other study reporting a median DFS of 24 months, most patients (81 %) also received preoperative chemotherapy [36]. Furthermore, the patients included in the mentioned study had a lower number of LM (1.2 per patient, with no patient having more than 2 LM) than the patients in our study. Noticeably, we were able to provide repeated curatively intended treatment for more than one third of the recurrent patients. However, this was only possible for those with oligometastatic recurrence.

In the literature, RFA has mostly been considered as a secondary treatment option compared to surgical resection or recommended as a supplement for hepatic resection [12, 37]. A study from 2016 reported a recurrence rate of LM in previously treated RFA cavities of 48 % with the highest risk for LM>3 cm and a margin size of ≤5 mm [38]. A meta-analysis from 2017 and a review from 2019 reported that RFA showed a higher recurrence rate, varying between 17 and 60 %, and a lower survival than resection [12, 13]. However, a study from 2022 performing propensity score matching on patients either undergoing RFA or resection for LM showed similar recurrence rates and survival [14]. In our study, we reported the LM recurrence rate in a previously RFA treated cavity to be 29 %, which is lower than recurrence rates reported in the previously mentioned studies. This may be due to the careful selection of patients.

Median LOS was ten days in the present study. Berger et al. reported a median LOS of eight days [39], but many other studies have reported a longer LOS, ranging from 12 to 23 days [36, 40, 41]. According to our guideline, until 2017 patients should be hospitalised for at least ten days. From 2017 onwards, mandatory LOS was reduced to seven days.

We reported a 30 day Clavien-Dindo ≥3 morbidity of 28 %. Berger et al. reported that 24 % developed Clavien-Dindo 3–4 complications within 30 days [39]. This is comparable to our findings. However, the proportion of patients requiring ICU admission in their study was 22 %, which is almost double the proportion reported in our study. The majority of other studies have reported a proportion of Clavien-Dindo ≥3 of 45–56 % [36, 40], i.e., up to two-fold higher than the findings of our study. Potentially, this higher proportion may impede or postpone the postoperative systemic chemotherapy that some patients require.

The present study is limited by its retrospective design. However, the data originate from a database with prospective data collection. We lack data on morbidity and LOS for a few patients. All 25 patients in the present study had undergone careful selection and thorough discussion at two MDT conferences before being assessed as eligible candidates. Despite only reporting on 25 patients, we believe that the reported data are relevant; and to our knowledge, the present study is the first to report on patients primarily treated with RFA.

The strength of this study is that we had survival data on each patient and recurrence data on all patients except one.

## Conclusions

The selected treatment modality (RFA) for CRC patients with both LM and PM was safe. Treating LM with RFA rather than liver resection may possibly yield a lower major 30 day postoperative morbidity rate, potentially allowing patients to proceed for postoperative chemotherapy. In the present study, DFS was relatively low compared to what has been reported in few previous studies. Further exploration in future studies is warranted to determine whether the lower DFS observed in the present study is due to resection being a more effective treatment for LM than RFA or if it is influenced by factors such as neoadjuvant treatment received by patients in other studies, potentially representing a more favourable patient population.
